# The Role of Zinc Supplementation in Alleviating Inflammatory Biomarkers in Patients Undergoing Hemodialysis: A Randomized Placebo Controlled, Crossover Trial

**DOI:** 10.5812/ijpr-147887

**Published:** 2024-09-15

**Authors:** Shayan Mastoor-Tehrani, Fariba Samadian, Hadi Esmaily, Alireza Kargar, Nasim Markazi, Shideh Anvari, Shadi Ziaie

**Affiliations:** 1 Department of Clinical Pharmacy, School of Pharmacy, Shahid Beheshti University of Medical Sciences (SBMU), Tehran, Iran; 2 Department of Nephrology and Kidney Transplantation, Shahid Labbafinejad Medical Center, Shahid Beheshti University of Medical Sciences, Tehran, Iran; 3 Department of Cardiology, Labbafinejad Hospital, Shahid Beheshti University of Medical Sciences, Tehran, Iran

**Keywords:** Zinc Deficiency, MHD, CRP, NLR, Quality of Life

## Abstract

**Background:**

Chronic kidney disease (CKD), which progresses to end-stage renal disease (ESRD) and requires maintenance hemodialysis (MHD), is a global health issue. Inflammation in MHD patients is associated with increased mortality and cardiovascular events. Zinc, essential for immune function and possessing anti-inflammatory properties, is frequently deficient in these patients and could potentially help mitigate inflammation.

**Objectives:**

This study aims to assess the impact of zinc supplementation on inflammatory biomarkers, specifically (CRP) and the neutrophil-to-lymphocyte ratio (NLR), in MHD patients.

**Methods:**

In a double-blind, randomized controlled crossover trial conducted at Labafinejad Hospital, Tehran, MHD patients with zinc deficiency were initially allocated to either a zinc supplementation group or a placebo group. After 30 days, the groups were crossed over, with patients initially receiving zinc now receiving a placebo and vice versa. The primary outcome was changes in serum zinc levels, while secondary outcomes focused on CRP and NLR levels.

**Results:**

Significant changes in serum zinc levels were observed in both groups from baseline to Month 2 (drug-placebo group: Mean change -15.9±10.33 µg/dL, P < 0.05; placebo-drug group: Mean change -14.70 ± 12.58 µg/dL, P < 0.05). A significant initial reduction in CRP levels at Month 1 (P = 0.045) was not sustained at Month 2 (P = 0.812). No statistically significant changes in NLR were noted. Improvements in quality of life, including reductions in muscle pain and skin dryness, were significant in the drug-placebo group (P < 0.05).

**Conclusions:**

Zinc supplementation in MHD patients significantly improved serum zinc levels and initially reduced CRP levels, highlighting its potential role in managing inflammation. Although the impact on NLR was not significant, overall patient outcomes and quality of life showed promising improvements.

## 1. Background

Chronic kidney disease (CKD) is a significant global public health challenge, affecting approximately 9 - 13% of the worldwide population (1, 2). A substantial proportion of CKD patients will eventually develop end-stage renal disease (ESRD), necessitating renal replacement therapy, including maintenance hemodialysis (MHD) (3). Chronic systemic inflammation in CKD and ESRD patients undergoing MHD is linked to increased cardiovascular events and mortality, and remains inadequately managed by current hemodialysis modalities (4). C-reactive protein (CRP) serves as a biomarker for chronic inflammation in patients undergoing hemodialysis (5). Additionally, the neutrophil-to-lymphocyte ratio (NLR) is recognized as another indicator of inflammation in those receiving dialysis treatment (6). Elevated levels of CRP and NLR correlate with a poor prognosis in patients undergoing MHD, indicating increased cardiovascular risk and predicting all-cause mortality in this population (6-9).

Zinc is an essential trace element involved in various physiological processes, including the regulation of immune function (10). The recommended daily allowance (RDA) for zinc is 11 mg for men and 8 mg for women (11). Zinc plays a critical role in managing ESRD by acting as both an antioxidant and an anti-inflammatory agent, helping to alleviate oxidative stress and inflammation—conditions common in ESRD patients. Additionally, zinc enhances immune function, which is often compromised in these individuals due to renal failure and the dialysis process. Studies have shown that zinc deficiency is common in ESRD due to reduced dietary intake, increased losses during dialysis, and impaired absorption. Supplementing zinc not only addresses this deficiency but may also improve overall health outcomes by mitigating complications associated with ESRD, such as infections, poor wound healing, and exacerbated cardiovascular disease risks (12-15). Research indicates that patients with ESRD, particularly those on MHD, exhibit reduced blood zinc levels, with the prevalence of zinc deficiency ranging between 40% and 78% (16, 17). Studies have suggested that zinc supplementation can modulate inflammatory cytokines and consequently reduce CRP levels (18-20), with observed reductions in serum CRP levels, especially in adults and pregnant women. In cases of chronic inflammatory diseases, zinc supplementation has been associated with notably lower CRP levels (20).

Certain studies suggest that zinc supplementation could potentially impact the neutrophil-to-lymphocyte ratio (NLR), although the data are less robust (21). While numerous studies have explored the role of zinc in modulating inflammatory markers in the general population (22, 23), there is a lack of comprehensive research specifically focusing on maintenance hemodialysis patients. Existing studies in this area have small sample sizes and vary in their methodological approaches (24).

Since inflammation plays a critical role in affecting the prognosis of MHD patients, and zinc has anti-inflammatory properties, this study is designed to evaluate the therapeutic potential of zinc supplementation for this patient group. We hypothesize that zinc supplementation will result in significant reductions in key inflammatory biomarkers, such as CRP and NLR, without causing adverse effects. By investigating the effects of zinc on these biomarkers, the study aims to address a gap in current medical research, potentially contributing to improved clinical outcomes and enhancing the quality of life for patients undergoing MHD. 

## 2. Objectives

This research may offer preliminary insights into the potential role of micronutrient supplementation in managing inflammation among MHD patients, cautiously suggesting its possible significance in this specific medical context.

## 3. Methods

### 3.1. Study Design and Setting

This research is designed as a double-blind, randomized controlled crossover clinical trial, conducted at Labafinejad Hospital in Tehran, Iran. The hospital's renowned nephrology and dialysis department is widely recognized for its medical services and patient care. The study adhered to the Consolidated Standards of Reporting Trials (CONSORT) guidelines (25, 26).

### 3.2. Participant Recruitment and Eligibility

Recruitment followed specific criteria to identify the relevant population. Inclusion criteria were individuals 18 years or older with confirmed zinc deficiency (serum zinc levels below 70 µg/dL) who had been receiving regular hemodialysis for at least six months, no less than twice a week. Exclusion criteria included lack of consent, hospitalization in the last three months, consumption of zinc or selenium supplements within 2 weeks before the study, significant co-morbidities, or medications affecting zinc metabolism or study outcomes.

### 3.3. Assessment Tool for Health-Related Quality of Life

In our research, we used the Persian version of the Kidney Disease Quality of Life – Short Form version 1.3 (KDQOL-SF™ 1.3) to evaluate health-related quality of life (HRQOL) in Iranian patients with end-stage renal disease (ESRD). This instrument was carefully adapted to fit the cultural and linguistic context of Iran, ensuring its relevance and reliability. Psychometric assessments demonstrated that the KDQOL-SF™ 1.3 has strong internal consistency (Cronbach’s alpha between 0.71 and 0.93) and test-retest reliability, with intraclass correlation coefficients exceeding 0.7 across all scales.

### 3.4. Sampling Method and Sample Size Calculation

The study employed simple randomization to select hemodialysis patients from Labafinejad Hospital, ensuring equal allocation to intervention or control groups and minimizing selection bias. Sample size calculation using G*Power was based on an effect size of 3, 80% power, and a 5% alpha level. Initially, 40 patients were selected, but the sample size was increased to 48 to account for a 20% attrition rate, thereby maintaining statistical power. This methodological approach enhances the reliability and validity of the study, contributing to a better understanding of treatment effects in hemodialysis patients.

### 3.5. Trial Phases and Intervention

The study was designed as a double-blind, randomized controlled crossover trial with two distinct 30-day phases. In the first phase, participants in the intervention group were administered a uniform dose of 137.3 mg of zinc sulfate daily (equivalent to 50 mg of elemental zinc). After completing this phase, participants immediately crossed over to receive the placebo treatment for an additional 30 days without a washout period. Conversely, the control group initially received a placebo, visually identical to the zinc supplement to ensure blinding but without the active ingredient, for the first 30 days, followed by the same dosage of zinc sulfate for the subsequent 30 days. The dosages of zinc sulfate and placebo were consistent across both phases of the trial.

Participants were instructed not to take any additional zinc supplements before and during the study period. Compliance was monitored through participant diaries and regular interviews, although strict controls over general dietary intake were not imposed.

Continuous safety monitoring was conducted, with regular assessments to detect adverse effects and ensure participant health.

During the trial, the primary outcomes measured were changes in C-reactive protein levels and the neutrophil-to-lymphocyte ratio. These inflammatory markers were evaluated to assess the impact of zinc supplementation on systemic inflammation in hemodialysis patients.

An initial screening and baseline assessment collected fasting blood samples to establish pre-intervention data on serum zinc levels, kidney function, nutritional and appetite status, and other clinical parameters. After the 60-day period, these measures were reassessed to evaluate the effects of zinc supplementation, focusing on serum zinc concentrations, inflammatory markers, and nutritional and appetite status.

### 3.6. Sequence Generation and Blinding

Participant assignment to intervention and control groups was achieved through simple randomization using a computer-generated sequence from sealedenvelope.com, ensuring unbiased distribution. Blinding was applied to participants, investigators, and outcome assessors to maintain objectivity. This strategy prevented allocation and observation biases, thereby enhancing the empirical validity and credibility of the study. A controlled unblinding process was in place for serious adverse events, ensuring ethical compliance while minimizing the impact on study integrity.

### 3.7. Anthropometric and Biochemical Assessments

Anthropometric and biochemical assessments were conducted at baseline and follow-up to evaluate participants. Measurements included body weight, height, BMI, and blood pressure. Biochemical evaluations focused on electrolytes (sodium, potassium, calcium, phosphorus) and other blood parameters, including complete blood count (CBC), platelets, and inflammatory markers. 

Data handling adhered to strict protocols for accuracy, integrity, and confidentiality. Advanced statistical methods were employed to ensure reliable findings. The study addressed challenges in measuring ESRD patients, such as body weight variations due to fluid balance. Informed consent was obtained, and protocols for managing adverse events were rigorously followed.

### 3.8. Primary and Secondary Endpoint and Outcome Measures

The primary endpoint evaluated changes in serum zinc levels and inflammatory markers from baseline to the end of the study in ESRD patients. The goal was to identify statistically and clinically significant improvements to support the effectiveness of zinc supplementation. Clinically meaningful improvements were defined using pre-established criteria, including specific numerical or percentage changes in serum zinc and other markers.

Secondary outcomes assessed the broader effects of zinc supplementation on patient health during hemodialysis, including overall well-being, with a particular focus on improvements in nutrition and appetite. Recognized tools and methods were used, including standardized questionnaires for well-being and quality of life. These assessments were aligned with the primary outcomes to ensure consistent data analysis.

### 3.9. Data Analysis and Statistical Management

The study used IBM SPSS Statistics software, version 27, for comprehensive data analysis. Data were systematically input, with each variable clearly defined. Robust multivariable adjustments were employed to isolate the effect of zinc supplementation on CRP levels, enhancing accuracy and validity.

Quantitative data were analyzed using means, medians, standard deviations, and ranges, while qualitative data were analyzed using frequency distributions and percentages.

Various statistical tests were applied, including ANOVA or *t*-tests for normally distributed data, chi-square tests for categorical data, and regression analysis (linear and logistic) as appropriate. Independent *t*-tests or Mann-Whitney U tests were used to compare continuous variables between groups, while paired *t*-tests or Wilcoxon signed-rank tests were used for within-group comparisons over time. Chi-square tests were used for categorical data analysis, and normality was assessed using the Kolmogorov-Smirnov and Shapiro-Wilk tests.

Imputation techniques, such as mean or regression imputation, were implemented to address missing data. The significance level was set at P < 0.05. A power analysis determined the necessary sample size to detect significant effects.

## 4. Results

This randomized, placebo-controlled, crossover trial rigorously evaluated the effects of zinc supplementation on inflammatory biomarkers and serum zinc levels in patients undergoing maintenance hemodialysis. The study was conducted over a period of six months, from October 2022 to April 2023. It was structured into two groups: The drug-placebo group and the placebo-drug group, each consisting of 20 participants.

### 4.1. Baseline Characteristics

Baseline characteristics, including age, gender distribution, and the etiology of chronic renal failure (CRF), are summarized in Table 1. These details ensure a well-balanced and comparable participant composition concerning these variables.

**Table 1.** Baseline Characteristics of Study Participants ^a^

Characteristic	Drug-Placebo Group (n = 20)	Placebo-Drug Group (n = 20)	P-Value
**Age (y)**	49.00 ± 11.3	54.73 ± 12.1	0.15
**Gender**			0.71
Female	6	4	
Male	14	16	
**Etiology of CRF**			
Diabetes	8	9	0.65
Hypertension	7	6	0.71
PKD	3	4	0.75
Others	2	1	0.81

^a^ Values are expressed as No. or mean ± SD.

### 4.2. Inflammatory Biomarkers

The analysis of inflammatory biomarkers, including CRP levels and NLR, across different time points is summarized in Table 2. 

**Table 2.** Comparative Analysis of Inflammatory Biomarkers ^a^

Biomarker and Measurement Point	Drug-Placebo Group	Placebo-Drug Group	P-Value
**CRP (mg/L)**			
Baseline	2.5 ± 1.0	2.3 ± 1.2	0.65
Month 1	1.8 ± 0.8	2.6 ± 1.0	0.045
Month 2	2.1 ± 0.9	2.2 ± 1.1	0.812
**NLR**			
Baseline	2.4 ± 0.9	2.5 ± 1.0	0.81
Month 1	2.2 ± 0.8	2.3 ± 1.1	0.75
Month 2	2.3 ± 0.9	2.4 ± 1.2	0.80

^a^ Values are expressed as mean ± SD.

### 4.3. Serum Zinc Levels

In this study, we examined the effects of zinc supplementation on serum zinc concentrations and CRP levels in hemodialysis patients. Statistically significant results were defined as those with a probability of less than 0.05. Both the drug-placebo and placebo-drug groups exhibited significant increases in serum zinc levels from baseline to the second month, with mean increases of 15.9 ± 10.33 µg/dL and 14.70 ± 12.58 µg/dL, respectively (P < 0.05). Linear regression analysis confirmed these changes, indicating a direct impact of the intervention.

In the drug-placebo group, serum zinc levels showed a statistically significant upward trend, with an initial level of 63.83 µg/dL and an average monthly increase of 7.95 µg/dL (P < 0.05). This consistent improvement underscores the efficacy of zinc supplementation in managing zinc deficiency in hemodialysis patients.

Logistic regression analysis revealed a significant association between zinc supplementation and CRP level improvement (OR = 2.45, 95% CI: 1.11 - 5.40, P = 0.03), controlling for baseline CRP levels. Patients receiving zinc had 2.45 times higher odds of CRP reduction compared to those on placebo. Baseline CRP levels also significantly predicted CRP improvement (P = 0.02), suggesting potential anti-inflammatory benefits of zinc supplementation.

Further logistic regression analysis, adjusting for additional confounders, did not find a significant association between zinc supplementation and variations in CRP levels (P > 0.05). This highlights the complexity of biochemical responses in hemodialysis patients and the importance of considering various physiological factors when evaluating nutritional interventions.

**Figure 1.**
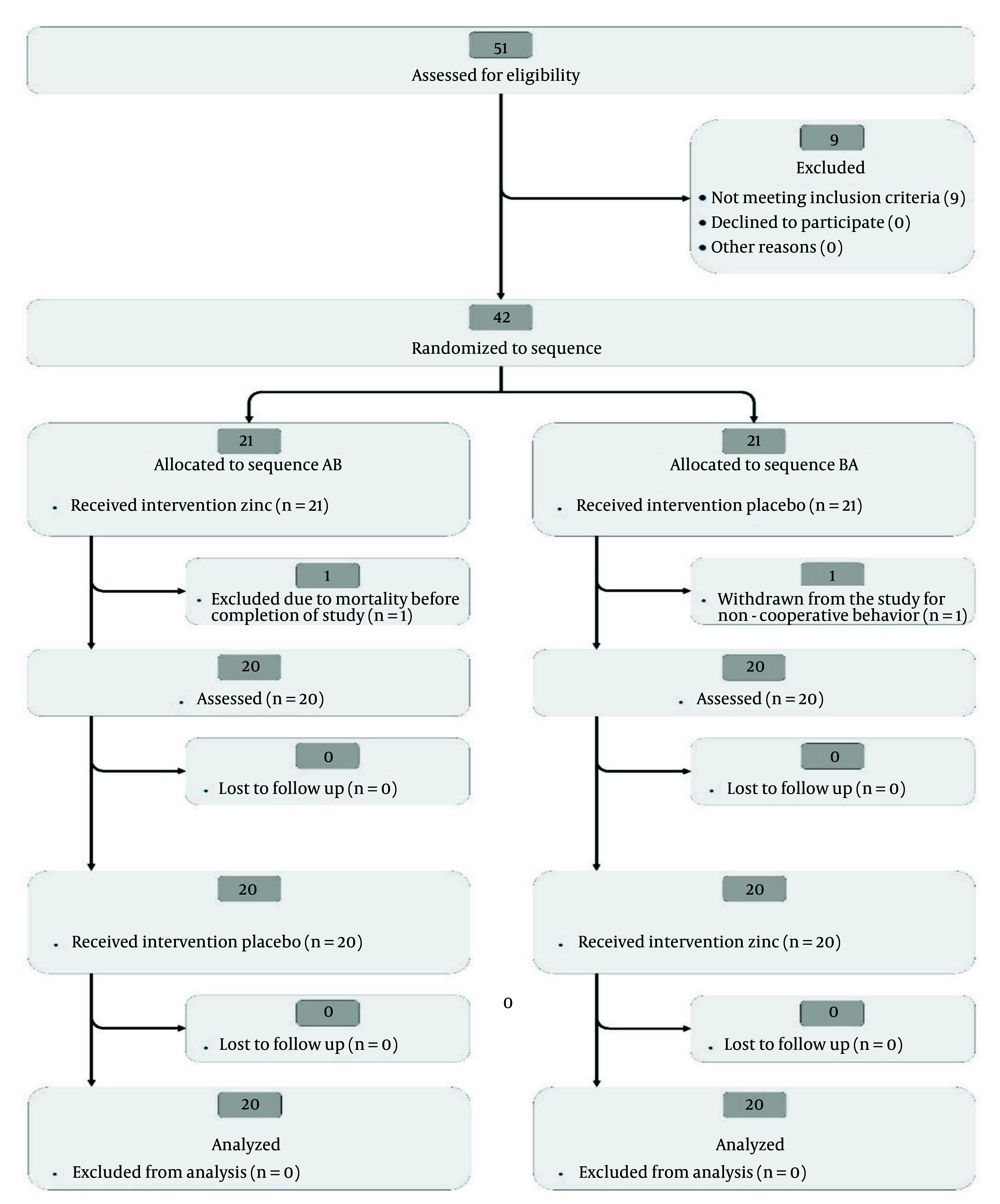
Consolidated Standards of Reporting Trials (CONSORT) flow diagram of participant progression through the phases of the randomized crossover trial

Overall, the significant changes in serum zinc levels from baseline to Month 2, detailed in Tables 3, and 4, support the reliability of our findings. The CONSORT flow diagram (Figure 1) illustrates the progression of participants through the study, underscoring the trial design's integrity.

**Table 3.** Comparative Analysis of Serum Zinc Levels Across Measurement Points

Measurement Point	Drug-Placebo Group	Placebo-Drug Group	Between Group Analysis P-Value
**Baseline**	61.65 ± 6.05 µg/dl	64.75 ± 3.73 µg/dl	0.23
**Month 1**	76.15 ± 14.80 µg/dl	62.5 ± 5.82 µg/dl	0.03
**Month 2**	77.55 ± 10.36 µg/dl	79.45 ± 13.21 µg/dl	0.57
**Within group analysis P-value**	< 0.05	< 0.05	

**Table 4.** Detailed Serum Zinc Level Changes by Group and Time Period ^a^

Group and Time Period	Values	STE	P-Value
Zinc			
**Drug-Placebo (µg/dL)**			
Baseline-M1	-14.50 ± 11.62	2.59	< 0.05
M1 - M2	-1.40 ± 17.23	3.85	0.720
Baseline-M2	-15.9 ± 10.33	2.31	< 0.05
**Placebo-Drug (µg/dL)**			
Baseline-M1	1.80 ± 5.14	1.15	0.134
M1 - M2	-16.50 ± 13.12	2.93	< 0.05
Baseline-M2	-14.70 ± 12.58	2.81	< 0.05

^a^ Values are expressed as No. or mean ± SD.

### 4.4. Other Biochemical Parameters

Electrolytes (Na, K, Ca, P), WBC, Neut, Lymph Counts, platelets, and Hemoglobin (Hg): No significant changes were observed between the groups or within groups in these counts and levels over the study period.

In addition to the primary clinical outcomes, this randomized controlled crossover trial also assessed the quality of life among participants in the drug-placebo and placebo-drug groups at different time points. Key findings, particularly those with statistically significant changes (P-Value < 0.05), are highlighted below:

### 4.5. Quality of Life Assessment

#### 4.5.1. Overall, Health

Overall health rating: In the drug-placebo Group, there was a significant improvement from Baseline to Month 1 (P = 0.008) and from Baseline to Month 2 (P = 0.014).

#### 4.5.2. Daily Activities

- Lifting or carrying groceries: The drug-placebo group showed no significant change from month 1 to month 2 (P = 0.564) and from baseline to month 2 (P = 0.564).

- Climbing several flights of stairs: In the placebo-drug group, there was no significant change from month 1 to month 2 (P = 0.083) and from baseline to month 2 (P = 0.083).

- Climbing one flight of stairs: The placebo-drug group showed no significant change from month 1 to month 2 (P = 0.180) and from baseline to month 2 (P = 0.180).

#### 4.5.3. Physical Health Impact on Activities

- Cut down time on work or other activities: The drug-placebo group showed no significant change from baseline to month 1 (P = 0.655) and from baseline to month 2 (P = 0.317).

- Accomplished less than liked: The placebo-drug group showed no significant change from baseline to month 1 (P = 0.083).

#### 4.5.4. Limitation in Work/Activities

- Drug-placebo group: No significant change was observed from baseline to month 1 (P = 0.564) or from baseline to month 2 (P = 1.000).

- Placebo-drug group: No significant change was observed from baseline to month 1 (P = 0.083) or from baseline to month 2 (P = 0.180).

#### 4.5.5. Emotional Problems Impacting Work/Activities

- Cut down time on work or other activities: In the placebo-drug group, there was no significant change from month 1 to month 2 (P = 0.083) or from Baseline to Month 2 (P = 0.083).

- Accomplished less than liked: The Placebo-drug group showed no significant change from month 1 to month 2 (P = 0.157) or from baseline to month 2 (P = 0.157).

- Didn't do work as carefully as usual: The placebo-drug group experienced a significant change from month 1 to month 2 (P = 0.046) and from baseline to month 2 (P = 0.046).

#### 4.5.6. Physical and Emotional Problems Interfering with Social Activities

Interference with social activities: The placebo-drug group showed no significant change from baseline to month 1 (P = 0.705) or from baseline to month 2 (P = 0.705).

#### 4.5.7. Physical Symptoms

Muscle pain: A significant change was observed in the drug-placebo group from baseline to month 1 (P = 0.021).

Skin dryness: Significant changes were observed in the drug-placebo group from baseline to month 1 (P = 0.003) and from baseline to month 2 (P = 0.012).

Loss of Appetite: Significant changes were observed in the drug-placebo group from Baseline to month 1 (P = 0.002), from month 1 to month 2 (P = 0.063), and from baseline to month 2 (P = 0.010).

#### 4.5.8. Emotional Well-being

Feeling nervous: No significant changes were observed in the drug-placebo group from baseline to month 1 (P = 0.414) or from baseline to month 2 (P = 0.334).

### 4.6. Safety and Tolerability

During the trial, two adverse events were reported in the group receiving zinc supplementation. The first involved a participant who developed skin lesions in the third week, identified as shingles, leading to the initiation of treatment. The second adverse event was dyspepsia, experienced by a participant in the first week of supplementation. Adjusting the supplement intake to one hour after meals successfully addressed this issue.

## 5. Discussion

This investigation highlights the potential role of zinc supplementation in modulating serum zinc concentrations and influencing inflammatory markers in hemodialysis patients. Initial changes in CRP levels were observed, but the enduring clinical significance of these effects requires further examination. Additionally, notable improvements were observed in various aspects of quality of life, particularly within the Drug-Placebo group. Improvements in muscle pain, skin dryness, appetite loss, and emotional well-being suggest that zinc supplementation has a positive impact on these facets of life. Comprehensive numerical and statistical analyses are provided in the accompanying tables and charts for a thorough understanding.

Zinc plays a crucial role in neurotransmitter regulation and protection against neurotoxicity, which are essential for cognitive functions and overall neurological health in patients with chronic kidney disease (CKD). It modulates the function of N-Methyl-D-aspartic acid (NMDA) and GABA receptors, which are vital for maintaining cognitive and psychological health. Zinc's interaction with these receptors helps balance excitatory and inhibitory signals in the brain, thereby impacting learning, memory, and mood regulation. Studies indicate that zinc's influence on these neurotransmitters can alleviate symptoms of depression and cognitive impairment, which are common in CKD patients. Moreover, zinc has neuroprotective properties that safeguard neurons from damage caused by oxidative stress and inflammatory cytokines. It helps regulate glutamate levels, preventing excitotoxicity—a process where excessive glutamate leads to neuronal injury and death. By mitigating oxidative stress and neuroinflammation, zinc contributes to better neuronal health and cognitive function. These neuroprotective and regulatory roles of zinc are directly linked to improving the quality of life in CKD patients by enhancing cognitive function, reducing depressive symptoms, and promoting overall neurological well-being (27-29).

Zinc deficiency in chronic kidney disease is attributed to several interrelated factors. Dietary restrictions imposed on CKD patients to manage electrolyte and fluid balance can inadvertently reduce zinc intake, as foods rich in zinc are often limited (30). Additionally, alterations in taste acuity, which are common in CKD, discourage adequate dietary intake and increase the risk of deficiency (31). Increased zinc losses during dialysis sessions are a significant contributor to zinc deficiency. Studies have quantified these losses, showing that hemodialysis can remove a substantial amount of zinc from the blood, which is not adequately replaced between sessions (32). Moreover, impaired gastrointestinal absorption of zinc in CKD patients, exacerbated by uremia and changes in the gut microbiome, affects zinc uptake (33-35). Finally, the chronic inflammatory state prevalent in CKD increases cytokine activity and metabolic stress, raising the body’s zinc requirements. This inflammatory response reduces plasma zinc levels through redistribution to the liver and inflammatory cells, a mechanism documented in inflammatory diseases (36).

Our research enhances the understanding of micronutrients, particularly zinc, in managing hemodialysis and its complications, aligning with existing literature. The 2021 meta-analysis by Mohammadi et al. highlighted zinc's ability to lower CRP, interleukin-6, and malondialdehyde levels, while increasing total antioxidant capacity among 1,428 hemodialysis patients, underscoring its anti-inflammatory and antioxidative properties (37). Similarly, our study observed initial anti-inflammatory responses with zinc supplementation. Jafari et al., in their systematic review and meta-analysis of 35 randomized controlled trials, reported significant reductions in CRP (weighted mean difference: -32.4) and hs-CRP (weighted mean difference: -0.95), alongside a decrease in neutrophil levels (standard mean difference: -0.46), indicating zinc's role in immune modulation (21). Mousavi et al. also supported zinc's efficacy in inflammation management, with their meta-analysis showing a weighted mean difference in CRP levels of -1.68 mg/l (95% CI: -2.4 to -0.9, P < 0.001) among patients with renal dysfunction (19).

Takic et al. highlighted zinc deficiency in hemodialysis patients, noting an average serum zinc concentration of 38.8 ± 7.72 μg/dL and a significant serum copper-to-zinc ratio of 2.76 ± 0.68, which correlated with increased CRP levels. This finding underscores the need for zinc supplementation to reduce inflammation (38). Furthermore, Ishioka et al. and Lobo et al. provided insights into zinc's role in cardiovascular health. They observed significantly lower plasma zinc levels in hemodialysis patients and a strong association between reduced zinc levels, increased lipid peroxidation, inflammation, and cardiovascular risk, with a 24.4% mortality rate due to cardiovascular disease in these patients. These findings highlight the critical role of zinc in managing CKD complications (39, 40).

The 2023 study by Ishioka et al. found that 57.0% of non-diabetic hemodialysis patients were zinc deficient (Zn < 60 μg/dL), which was associated with higher CRP levels and increased arterial stiffness, as measured by brachial-ankle pulse wave velocity (baPWV). This suggests that zinc deficiency is an independent risk factor for increased arterial stiffness (39).

Building on the role of zinc in cardiovascular health, the 2017 study by Hosseinzadeh-Attar et al. observed differences in Zinc-alpha2-glycoprotein (ZAG) levels between normal-weight and obese hemodialysis patients (100 ± 34 ng/mL vs. 106 ± 31 ng/mL; P = 0.007). This indicated a relationship between zinc metabolism, obesity, and inflammation, as evidenced by higher LDL/HDL ratios and elevated hsCRP levels in obese patients (41).

In 2022, Hosseini et al. conducted a randomized, double-blind, placebo-controlled trial with 46 diabetic hemodialysis patients who were zinc deficient. Zinc sulfate supplementation (220 mg/day, containing 50 mg of elemental zinc) significantly increased serum zinc levels from 55.9 ± 8.02 to 90.6 ± 15.71 mg/dL (P < 0.001) and reduced high-sensitive CRP (hs-CRP) from 8.73 ± 4.2 mg/L to 4.92 ± 2.07 mg/L (P < 0.001), indicating a decrease in inflammation. Additionally, improvements were noted in renal function markers, fasting blood glucose, BMI, and body weight, with no significant changes in serum insulin levels or insulin sensitivity indicators (42).

Guo et al. observed significantly lower zinc levels in hemodialysis patients (0.36 ± 0.04 μg/mL) compared to those undergoing peritoneal dialysis (PD) (0.68 ± 0.03 μg/mL) and healthy controls (0.76 ± 0.08 μg/mL), with elevated CRP levels in HD patients (15.71 ± 2.03 ng/mL vs. 8.42 ± 1.14 ng/mL in PD and 0.68 ± 0.12 ng/mL in controls), suggesting a link between zinc deficiency and inflammation (43).

The 2019 study by Escobedo-Monge et al. (24) involving 48 children with CKD who received zinc supplementation (30 or 15 mg/day) for a year showed no significant changes in serum albumin, zinc, and CRP levels. However, a significant positive correlation between serum zinc concentration (SZC) and serum albumin was observed both before (r = 0.64; P = 0.000) and after zinc supplementation (r = 0.55; P = 0.007), with a modest improvement in BMI Z-score at the 30 mg/day dosage, indicating potential nutritional benefits (24).

The KDIGO, NKF KDOQI, CMS ESRD QIP, and ERBP guidelines all provide comprehensive recommendations for managing chronic kidney disease, dialysis, and end-stage renal disease (ESRD). However, specific guidance on zinc supplementation is not prominently detailed in these guidelines. The KDIGO and ERBP guidelines focus on broader aspects of CKD management without explicit recommendations for zinc supplementation. The NKF KDOQI guidelines acknowledge the importance of achieving adequate dietary intake of essential minerals, including zinc, but do not provide detailed protocols specifically for CKD or dialysis patients. The CMS ESRD QIP emphasizes quality improvement and patient outcomes but does not address zinc supplementation specifically (44-47).

These findings collectively highlight the multifaceted role of zinc in hemodialysis patients, demonstrating its potential in reducing inflammation and improving cardiovascular health, particularly among those with additional complications such as obesity and diabetes.

In summary, the extensive body of research, exemplified by studies from Zhang et al. (48), Wang et al. (49), Al-Hakeim et al. (50), Aranha et al. (51), Liu et al. (52), Ribeiro et al. (53), and Dizdar et al. (54), reinforces the multifaceted role of zinc in managing various health aspects in patients undergoing maintenance hemodialysis. Zhang et al. (48) highlighted a significant prevalence of coronary artery calcification (CAC) in CKD patients, linking lower zinc levels (below 86.62 μmol/L) to an elevated risk of cardiovascular events. This is crucial given the 58.82% prevalence of CAC in their study group. Their findings underscore the inverse relationship between whole blood zinc levels and CAC severity, emphasizing the need to monitor zinc levels for cardiovascular risk assessment in these patients.

Wang et al. (49), in their 2020 research, examined altered mineral metabolism in chronic hemodialysis patients, noting significantly lower zinc levels (0.7 μg/mL compared to 0.9 μg/mL in controls) and elevated copper and magnesium levels. They associated these imbalances with increased oxidative stress and inflammation, evidenced by higher malondialdehyde levels (5.0 nmol/L vs. 2.3 nmol/L in controls) and CRP levels (10.5 mg/L vs. 1.0 mg/L in controls). This study highlights the complex interplay between mineral metabolism, oxidative stress, and inflammation, which may contribute to accelerated vascular calcification in hemodialysis patients.

Al-Hakeim et al. (50) found a strong correlation between neuropsychiatric symptoms, kidney dysfunction, and dialysis frequency in ESRD patients. They observed elevated CRP levels (7.51 mg/L) and reduced serum zinc levels (0.67 mg/L), linking neurotoxicity and inflammation to these symptoms. Aranha et al. (51) reported high leptin levels (16.1 μg/mL) and low serum zinc levels (54.5 μg/dL) in hemodialysis patients, with a negative correlation between leptin and zinc levels (r = -0.33; P = 0.007) and inflammatory markers, suggesting interactions between micronutrient status, inflammation, and hormonal regulation.

Liu et al. associated blood zinc levels below 4.220 mg/L with an increased risk of malnutrition (OR = 3.723; P = 0.016) and found that low blood zinc levels were independently associated with a higher nutritional risk (OR = 0.634; P = 0.015) (52). Ribeiro et al. linked impaired appetite in hemodialysis patients to lower zinc intake (OR = 0.860; p = 0.03), higher body fat percentages, and reduced zinc intake, indicating a connection between zinc deficiency and appetite (53). Dizdar et al. observed a significant prevalence of zinc deficiency in ESRD patients on hemodialysis, with 44.1% showing low zinc levels and elevated CRP levels. This suggests a relationship between inflammation and zinc deficiency, although no significant changes in zinc levels were observed over six months (54).

These studies collectively support the role of zinc supplementation as a crucial part of treatment for hemodialysis patients, highlighting its importance in managing inflammation, improving quality of life, and addressing cardiovascular risks.

### 5.1. Study Limitations

Although the results of this study are promising, there are several limitations to consider. The sample size was relatively small, which may affect the generalizability of our findings. The short duration of the study limited our ability to evaluate the long-term effects of zinc supplementation. Additionally, despite controlling for dietary zinc intake, achieving full dietary control is challenging, and there remains the possibility of residual confounding by other dietary elements. To verify and expand upon these findings, future research should include larger sample sizes and longer follow-up periods.

### 5.2. Conclusions

This study provides significant evidence that zinc supplementation in patients undergoing maintenance hemodialysis due to chronic kidney disease and end-stage renal disease leads to a notable reduction in key inflammatory biomarkers, specifically C-reactive protein and the neutrophil-to-lymphocyte ratio. Our findings suggest that addressing zinc deficiency in this patient group not only mitigates inflammation but also holds potential for improving overall patient outcomes. This research offers valuable insights into the therapeutic role of micronutrients in managing chronic conditions associated with MHD, presenting a promising avenue for enhancing patient care in this context.

## Data Availability

The dataset presented in the study is available on request from the corresponding author during submission or after publication.

## Clinical Trial Registration Code:

This clinical trial was registered with the Iranian Registry of Clinical Trials under the code IRCT20160412027346N8.
